# The role of the duration of untreated illness (DUI) in generalized anxiety disorder: a cross-sectional, multicenter study

**DOI:** 10.1017/S1092852925100758

**Published:** 2026-01-12

**Authors:** Letizia Maria Affaticati, Elisa Giglio, Enrico Capuzzi, Irene Riva, Davide La Tegola, Fabrizia Colmegna, Massimo Clerici, Massimiliano Buoli

**Affiliations:** 1 Department of Medicine and Surgery, University of Milano-Bicocca, Italy; 2 Mental Health, Fondazione IRCCS San Gerardo dei Tintori, Italy; 3 Universita degli Studi di Milano, Italy

**Keywords:** Duration of untreated illness, GAD, generalized anxiety disorder, outcome

## Abstract

**Objective:**

A longer duration of untreated illness (DUI) has been associated with poorer outcomes across several mental disorders; however, few studies have investigated DUI in anxiety disorders, particularly in generalized anxiety disorder (GAD). This study aimed to identify sociodemographic and clinical factors associated with a longer DUI in GAD.

**Methods:**

We conducted a cross-sectional, multicenter study, retrospectively reviewing the medical records of GAD patients from three mental health services. Sociodemographic and clinical variables were extracted for analysis. One-way analyses of variance and Pearson’s correlations were used to examine the relationship between DUI and categorical and quantitative variables, respectively. A multivariate linear regression model was then conducted to identify variables independently associated with DUI.

**Results:**

The total sample included 243 patients; the mean DUI was 30.92 (±65.25) months. In the final model, a longer DUI was associated with an earlier age at onset (*B* = −0.428; *p* = 0.023), a longer duration of illness (*B* = −0.431; *p* < 0.001), and the presence of multiple side effects (*B* = 55.778; *p* < 0.001). There was a trend toward statistical significance for the association between a longer DUI and multiple medical comorbidities (*B* = 13.122; *p* = 0.076).

**Conclusions:**

Our findings suggest that reducing the time between the onset of GAD and the initiation of appropriate pharmacological treatment may improve clinical outcomes, mitigating the risk of a chronic course of illness. Further studies are needed to elucidate the role of DUI as a prognostic factor in GAD.

## Introduction

Generalized anxiety disorder (GAD) is a common mental health condition with a lifetime prevalence of 12.1%,^1^ imposing a significant economic burden on healthcare systems.^2^ GAD is characterized by unfocused and excessive worry, and uncontrollable anxiety without a direct connection to recent stressful events.^3^ In its severe expressions, the condition may lead to social and occupational impairment,^4^ poor quality of life,^5^ and suicidal behavior.^6^ GAD often occurs in comorbidity with other mental health conditions, particularly major depressive disorder and other anxiety disorders, which may complicate its manifestations, aggravate its course, and contribute to a worse treatment response.^7^^,^^8^

According to international guidelines, selective serotonin reuptake inhibitors (SSRIs), serotonin and noradrenaline reuptake inhibitors (SNRIs), and pregabalin should be employed as first-line agents in the treatment of GAD.^9^^,^^10^ Benzodiazepines, such as diazepam, are suggested as secondary alternatives for initial symptom control, in combination with the primary treatment.^11^ Moreover, some clinical trials have reported a beneficial effect for agomelatine, mood stabilizers, and atypical antipsychotics, either as monotherapy or as augmentation to standard pharmacotherapy.^12^^–^^14^ Despite the availability of effective pharmacological and psychosocial approaches,^15^ only an estimated 43.2% of individuals with GAD receive any form of treatment.^16^

The duration of untreated illness (DUI) is defined as the interval between the onset of a disorder and the administration of the first approved pharmacological treatment, at standard dosages and for an appropriate duration.^17^ A growing number of studies suggest that DUI can modify the course of various psychiatric conditions: a longer delay to treatment appears to be associated with increased suicide attempts, more frequent hospitalizations, and poorer treatment response.^18^^–^^20^ While most studies have focused on the role of DUI in psychotic and affective disorders, research across anxiety disorders remains comparatively limited. However, preliminary findings, particularly in panic disorder, suggest that this area warrants further investigation.^21^^,^^22^ Among anxiety disorders, patients with GAD are reported to experience the longest delay to pharmacological treatment.^23^^,^^24^ Despite this, only one study to date^25^ has thoroughly examined the relationship between DUI and clinical factors in patients affected by GAD. The study found that a longer latency to treatment was associated with poorer response to pharmacotherapy, prolonged illness duration, and the development of comorbid mood and anxiety disorders.^25^ Albeit limited, these findings suggest that a more comprehensive understanding of clinical variables associated with DUI could ameliorate the management of GAD patients. Therefore, the objective of the present study was to identify sociodemographic and clinical factors associated with a longer DUI in GAD.

## Materials and methods

### Study design and patient’s sample

We conducted a cross-sectional, multicenter study. Outpatients diagnosed with GAD were retrospectively identified from medical records compiled during the pre-pandemic period (2016–2019) at three Italian mental health services: Fondazione IRCCS San Gerardo dei Tintori (Monza), Fondazione IRCCS Ca′ Granda, Ospedale Maggiore Policlinico (Milan), and Ospedale Treviglio-Caravaggio (Bergamo). GAD diagnoses were made by a senior psychiatrist based on the criteria outlined in the Diagnostic and Statistical Manual of Mental Disorders—5th edition (DSM-5, APA 2013). At the time of the first psychiatric encounter, when medical records were compiled, clinical information was gathered through patient and relative interviews and by consulting the psychiatric database of the Lombardia Region.

Inclusion criteria were: (1) GAD as the main psychiatric diagnosis; (2) fluency in Italian; (3) ability and willingness to sign informed consent; and (4) a minimum treatment duration of 3 months at the outpatient clinic. Patients were excluded on the following grounds: (1) age < 18 years; (2) intellectual disability; (3) other DSM-5 diagnoses of anxiety disorders; and (4) insufficient sociodemographic and clinical information.

The following demographic and clinical variables were extracted from medical records for analysis: sex, age, occupational and marital status, age at onset, duration of illness, DUI (months), presence and type of psychiatric and medical comorbidities before and after GAD onset, cluster of comorbid personality disorder (PD), substance use disorders before and after GAD onset, family history of mental disorders, suicide attempts, hospitalizations, presence and type of obstetric complications, primary pharmacological treatment, poly-therapy, side effects, presence of multiple side effects, treatment duration (months), reason for treatment discontinuation, and the presence and type of lifetime psychotherapy. DUI was defined as the time elapsed between the onset of GAD and the initiation of the first appropriate pharmacological treatment, at standard dosages and for an appropriate duration, excluding benzodiazepines.^23^^,^^26^

Study procedures adhered to the provisions of the Declaration of Helsinki, and the research project was reviewed and approved by the local accredited Medical Ethics Review Committee (Area 2 Ethics Committee) of Fondazione IRCCS San Gerardo dei Tintori, Monza (approval code 3955).

### Statistical analyses

Descriptive analyses were carried out on the whole sample. Frequencies with percentages were reported for categorical variables, and means and standard deviations were calculated for quantitative variables.

One-way analyses of variance (ANOVAs) and Pearson’s correlations were conducted to assess the relationship between DUI and categorical and quantitative variables, respectively. To identify the most relevant set of variables independently associated with the length of DUI, a linear multivariate regression model (backward procedure) was then employed, including variables that were statistically significant in univariate analyses.

Statistical significance was set at a nominal *p*-value of ≤0.05. The aforementioned analyses were conducted using the Statistical Package for Social Sciences for Windows (version 27.0).

## Results

### Descriptive analyses

A total of 243 individuals with a GAD diagnosis were included. Among these, 162 (66.7%) were female, and 81 (33.3%) were male. The mean age of the sample was 47.83 (±16.84) years, and the mean DUI was 30.92 (±65.25) months. Results of descriptive and univariate analyses of categorical variables are presented in Tables 1–4 and the corresponding results for quantitative variables are presented in Table 5.

**Table 1.** Descriptive Statistics for the Total Sample (*N* = 243) on Sociodemographic and Family History Categorical Variables, and Corresponding Results of Univariate Analyses

Variables		*N*	%	DUI (mean ± SD)	*p*-values
Sex	Female	162	66.7	34.12 ± 72.84	0.280
	Male	81	33.3	24.52 ± 46.23	
Work	Employed	200	82.3	31.08 ± 69.07	0.886
	Unemployed	37	15.2	32.16 ± 46.73	
	Never employed	6	2.5	18.17 ± 15.78	
Marital status	Single	79	32.5	27.46 ± 46.13	0.830
	In a relationship	136	56.0	33.52 ± 77.15	
	Divorced	25	10.3	30.76 ± 48.63	
	Widowed	3	1.2	5.67 ± 8.08	
Family history of mental disorders (dichotomous)	No	145	59.7	30.20 ± 70.93	0.834
	Yes	98	40.3	31.99 ± 56.13	
Family history of mental disorders	None	145	59.7	30.20 ± 70.93	0.855
	Psychotic disorders	48	19.8	26.08 ± 46.38	
	Bipolar disorder	8	3.3	40.50 ± 76.08	
	MDD	25	10.3	34.56 ± 54.33	
	GAD	12	4.9	54.25 ± 86.29	
	Others	2	0.8	11.50 ± 9.19	
	Substance misuse	3	1.2	7.00 ± 5.57	
Family history of multiple mental disorders	Yes	19	7.8	27.05 ± 30.96	0.788
	No	224	92.2	31.25 ± 67.39	

Abbreviations: DUI, duration of untreated illness; GAD, generalized anxiety disorder; MDD, major depressive disorder; SD, standard deviation.

Statistically significant results are shown in bold.

**Table 2.** Descriptive Statistics for the Total Sample (*N* = 243) on Mental Health Categorical Variables, and Corresponding Results of Univariate Analyses

Variables		*N*	%	DUI (mean ± SD)	*p*-values
Pre-onset psychiatric diagnosis (dichotomous)	No	157	64.4	29.56 ± 64.35	0.661
	Yes	86	35.4	33.41 ± 67.16	
Pre-onset psychiatric diagnosis	None	157	64.6	29.56 ± 64.35	0.875
	Mood episode	43	17.7	31.35 ± 64.35	
	Panic disorder	9	3.7	44.89 ± 77.91	
	OCD	2	0.8	88.00 ± 124.45	
	ED	8	3.3	16.38 ± 21.89	
	PTSD	2	0.8	30.00 ± 16.97	
	Other	22	9.1	34.27 ± 81.98	
Post-onset psychiatric comorbidity (dichotomous)	No	164	67.5	25.78 ± 57.85	0.077
	Yes	79	32.5	41.59 ± 77.73	
Post-onset psychiatric comorbidity	None	164	67.5	25.78 ± 57.85	0.652
	Mood episode	39	16.0	25.21 ± 47.75	
	PD	5	2.1	168.60 ± 151.56	
	OCD	2	0.8	88.00 ± 124.45	
	ED	7	2.9	18.00 ± 23.12	
	Other	26	10.7	44.54 ± 83.54	
Presence of multiple pre-onset psychiatric diagnoses	No	232	95.5	31.48 ± 66.65	0.542
	Yes	11	4.5	19.18 ± 15.33	
Presence of multiple post-onset psychiatric comorbidity	No	224	92.2	30.71 ± 66.12	0.865
	Yes	19	7.8	33.37 ± 55.37	
Addiction or substance misuse before GAD onset (dichotomous)	No	222	91.4	31.73 ± 67.37	0.529
	Yes	21	8.6	22.33 ± 35.66	
Addiction or substance misuse before GAD onset	None	222	91.4	31.73 ± 67.37	0.963
	Alcohol	8	3.3	21.88 ± 25.53	
	Cannabis	7	2.9	13.43 ± 15.50	
	Cocaine	3	1.2	56.67 ± 86.17	
	Opioids	1	0.4	12.00	
	BDZ	1	0.4	12.00	
	Gaming addiction	1	0.4	6.00	
Addiction or substance misuse after GAD onset (dichotomous)	No	230	94.7	30.02 ± 65.05	0.367
	Yes	13	5.3	46.85 ± 69.33	
Addiction or substance misuse after GAD onset	None	230	94.7	30.02 ± 65.05	0.406
	Alcohol	6	2.5	87.00 ± 87.50	
	Cannabis	3	1.2	18.33 ± 24.21	
	Cocaine	1	0.4	2.00	
	BDZ	2	0.8	12.00 ± 0.00	
	Gaming addiction	1	0.4	6.00	
Poly-substance misuse before GAD onset	No	234	96.3	31.66 ± 66.36	0.371
	Yes	9	3.7	11.78 ± 8.55	
Poly-substance misuse after GAD onset	No	240	98.8	31.27 ± 65.58	0.457
	Yes	3	1.2	3.00 ± 2.65	
Cluster of comorbid personality disorders (dichotomous)	No	210	86.4	29.02 ± 64.77	0.253
	Yes	33	13.6	43.00 ± 67.96	
Cluster of comorbid personality disorders	None	210	86.4	29.02 ± 64.77	**0.024**
	Cluster A	5	2.1	11.20 ± 20.72	
	Cluster B	20	8.2	26.20 ± 33.53	
	Cluster C	5	2.1	100.40 ± 126.73	
	NOS	3	1.2	112.33 ± 97.16	
Previous suicide attempts	No	235	96.7	31.25 ± 66.17	0.671
	Yes	8	3.3	21.25 ± 26.50	
Previous hospitalizations	No	225	92.6	31.56 ± 66.08	0.588

Abbreviation: BDZ, benzodiazepine; DUI, duration of untreated illness; ED, eating disorder; GAD, generalized anxiety disorder; NOS, not otherwise specified; OCD, obsessive compulsive disorder; PD, personality disorder; PTSD, post-traumatic stress disorder; SD, standard deviation.

Statistically significant results are shown in bold.

**Table 3.** Descriptive Statistics for the Total Sample (*N* = 243) on Categorical Variables Related to Medical Comorbidities and Obstetric History, and Corresponding Results of Univariate Analyses

Variables		*N*	%	DUI (mean ± SD)	*p*-values
Diagnosis of medical illness before GAD onset (dichotomous)	No	133	54.7	33.20 ± 67.45	0.550
	Yes	100	41.2	28.16 ± 62.67	
Diagnosis of medical illness before GAD onset	None	133	54.7	33.20 ± 67.45	0.867
	Obesity	5	2.1	23.60 ± 22.49	
	Hypercholesterolemia	6	2.5	74.67 ± 70.28	
	Hypertension	22	9.1	43.59 ± 96.14	
	Diabetes	5	2.1	34.80 ± 67.85	
	Hyperthyroidism	2	0.8	12.00 ± 0.00	
	Hypothyroidism	8	3.3	42.25 ± 55.36	
	Stroke	2	0.8	9.00 ± 12.73	
	Epilepsy	2	0.8	24.00 ± 33.94	
	Migraine	8	3.3	8.13 ± 8.51	
	Gastroenteric illness	18	7.4	31.39 ± 85.14	
	Neoplasia	9	3.7	13.89 ± 24.58	
	Ischemic heart disease	2	0.8	6.00 ± 8.48	
	Asthma	2	7.8	13.00 ± 15.56	
	Other	19	0.8	9.37 ± 11.41	
Diagnosis of medical illness after GAD onset (dichotomous)	No	134	55.1	31.65 ± 64.56	0.848
	Yes	109	44.9	30.03 ± 66.37	
Diagnosis of medical illness after GAD onset	None	134	55.1	31.65 ± 64.55	0.905
	Obesity	5	2.1	22.40 ± 23.77	
	Hypercholesterolemia	8	3.3	46.50 ± 52.28	
	Hypertension	29	11.9	47.28 ± 103.51	
	Diabetes	7	2.9	27.43 ± 56.84	
	Hyperthyroidism	1	0.4	1.00	
	Hypothyroidism	10	4.1	38.00 ± 50.62	
	Epilepsy	1	0.4	0.00	
	Migraine	10	4.1	7.80 ± 7.97	
	Gastroenteric illness	14	5.8	33.64 ± 77.47	
	Neoplasia	4	1.6	1.50 ± 1.73	
	Asthma	2	0.8	13.00 ± 15.56	
	Other	18	7.4	14.67 ± 19.37	
Presence of multiple medical illnesses before GAD onset	No	191	78.6	31.46 ± 66.53	0.806
	Yes	52	21.4	28.94 ± 60.85	
Presence of multiple medical illnesses after GAD onset	No	188	77.4	25.89 ± 54.90	**0.026**
	Yes	55	22.6	48.13 ± 90.83	
Obstetrical complications	No	234	96.3	31.50 ± 66.36	0.486
	Yes	9	3.7	16.00 ± 16.22	
Type of obstetrical complication	None	234	96.3	31.50 ± 66.36	0.753
	Low birth weight	4	1.6	23.00 ± 23.31	
	Gestational age < 37 weeks	5	2.1	10.40 ± 5.55	

Abbreviation: DUI, duration of untreated illness; GAD, generalized anxiety disorder; SD, standard deviation.

Statistically significant results are shown in bold.

**Table 4.** Descriptive Statistics for the Total Sample (*N* = 243) on Categorical Treatment Variables, and Corresponding Results of Univariate Analyses

Variables		*N*	%	DUI (mean ± SD)	*p*-values
Pharmacological treatment (dichotomous)	No	12	4.9	51.67 ± 41.71	0.259
	Yes	231	95.1	29.84 ± 66.12	
Main pharmacological treatment	None	12	4.9	51.67 ± 41.71	0.873
	Multimodal AD	11	4.5	19.09 ± 25.14	
	SNRI	24	9.9	25.71 ± 27.78	
	SSRI	142	58.4	31.81 ± 72.52	
	TCA	4	1.6	59.25 ± 70.86	
	Other ADs	3	1.2	48.00 ± 72.99	
	GABAergic compound	13	5.3	42.54 ± 110.30	
	SGA	10	4.1	18.30 ± 33.11	
	Mood stabilizer	1	0.4	43.00	
	BDZ	23	9.5	16.96 ± 44.92	
Presence of poly-therapy	No	105	43.2	23.03 ± 41.63	0.100
	Yes	138	56.8	36.93 ± 78.23	
Side effects (dichotomous)	No	217	89.3	29.64 ± 62.89	0.378
	Yes	26	10.7	41.62 ± 83.07	
Side effects	None	217	89.3	29.64 ± 62.89	0.526
	Weight gain	5	2.1	91.00 ± 164.88	
	Somnolence	11	4.5	28.64 ± 41.20	
	Low sex drive	2	0.8	7.00 ± 7.07	
	Nausea	2	0.8	40.50 ± 27.58	
	Anxiety	2	0.8	10.00 ± 11.31	
	Others	4	1.6	49.25 ± 95.17	
Presence of multiple side effects	No	239	98.4	29.02 ± 60.70	**0.000**
	Yes	4	1.6	144.75 ± 183.04	
Reason for treatment discontinuation	None	207	85.2	31.05 ± 67.82	0.240
	Symptom remission	13	5.3	3.85 ± 3.98	
	Side effects	7	2.9	24.86 ± 48.05	
	Relapse	9	3.7	69.78 ± 70.56	
	No *compliance*	7	2.9	33.43 ± 29.73	
Lifetime psychotherapy	Yes	67	27.6	28.70 ± 41.17	0.744
	No	176	72.4	31.77 ± 72.42	
Type of psychotherapy	None	176	72.4	31.77 ± 72.42	0.943
	Psychoeducation	13	5.3	28.00 ± 30.63	
	CBT	33	13.6	25.18 ± 38.92	
	Psychodynamic	15	6.2	25.67 ± 22.17	

Abbreviation: AD, antidepressant; BDZ, benzodiazepine; CBT, cognitive behavioral therapy; DUI, duration of untreated illness; ED, eating disorder; GAD, generalized anxiety disorder; SD, standard deviation; SGA, second-generation antipsychotic; SNRI, serotonin-norepinephrine reuptake inhibitor; SSRI, selective serotonin reuptake inhibitor; TCA, tricyclic antidepressant.

Statistically significant results are shown in bold.

**Table 5.** Pearson’s Correlations Between DUI and Quantitative Variables

Variables	Mean	SD	Pearson’s correlation (*r*)	*p*-values
Age (years)	47.83	16.843	0.101	0.118
Age at onset (years)	40.77	16.792	−0.238	**<0.001**
Duration of illness (months)	90.21	98.822	0.708	**<0.001**
Number of suicide attempts	0.05	0.349	−0.028	0.660
Number of hospitalizations	0.1	0.394	−0.051	0.428
Treatment duration (months)	44.96	52.911	0.081	0.209

Abbreviation: DUI, duration of untreated illness; SD, standard deviation. Statistically significant results are shown in bold.

### One-way ANOVAs and Pearson’s correlations

Patients with multiple medical illnesses occurring after GAD onset (*F* = 5.026; *p* = 0.026) and those with multiple side effects from pharmacological treatment (*F* = 12.992; *p* < 0.001) had a longer DUI compared to their counterparts. Moreover, DUI length was significantly different based on the type of psychiatric comorbidity commencing after GAD onset (*F* = 5.833; *p* < 0.001), as well as the cluster of comorbid PD (*F* = 2.855; *p* = 0.024). In particular, post-hoc analyses showed that DUI was significantly longer in subjects who developed a panic disorder compared to those developing a major mood episode (*p* < 0.001), an eating disorder (*p* = 0.001), other psychiatric disorders (*p* = 0.001), or no psychiatric comorbidities (*p* < 0.001) after GAD onset. In addition, individuals with a ‘Cluster C’ or ‘Not otherwise specified’ (NOS) PD had a longer DUI compared to those with other clusters or no PDs (*p* = 0.024). No significant differences were found among patient groups based on other categorical variables.

A significant inverse correlation was found between DUI length and age at onset (*r* = −0.238; *p* < 0.001), and a significant direct correlation was observed between DUI length and duration of illness (*r* = 0.708; *p* < 0.001). No other significant results emerged for quantitative variables.

### Linear multivariate regression model

**Figure 1.**
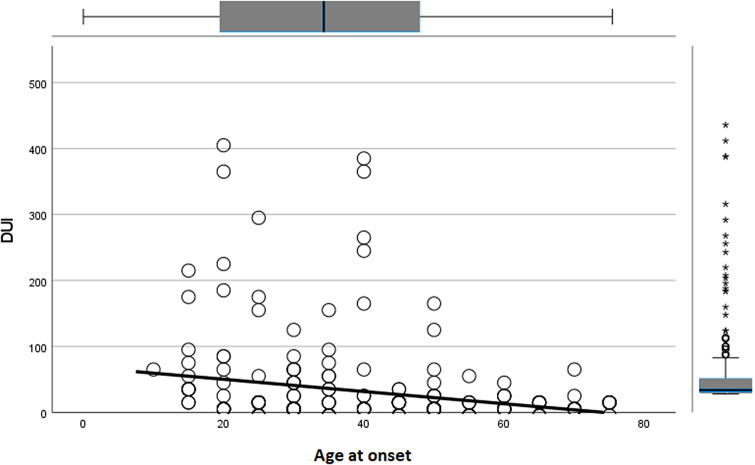
Significant association between age at onset and length of DUI (months) in GAD patients. Abbreviation: DUI, duration of untreated illness. Statistics: *B* = −0.428; *p* = 0.023.

**Figure 2.**
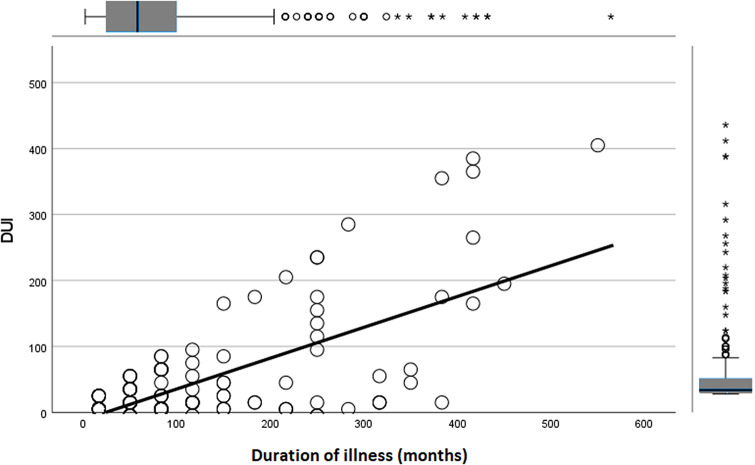
Significant association between duration of illness and length of DUI in GAD patients. Abbreviation: DUI, duration of untreated illness. Statistics: *B* = 0.431; *p* < 0.001.

**Figure 3.**
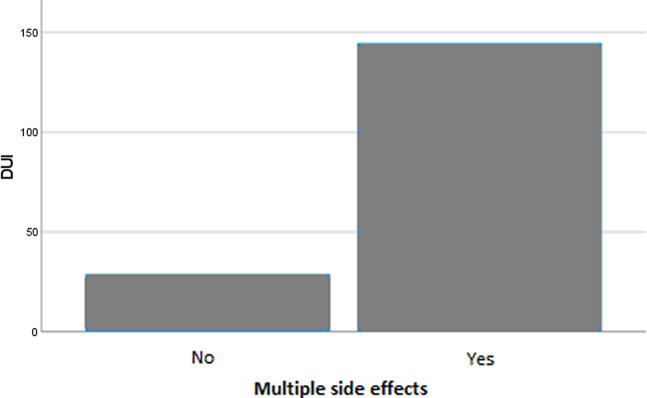
Significant association between the length of DUI (months) and the presence of multiple side effects in GAD patients. Abbreviation: DUI, duration of untreated illness. Statistics: *B* = 2.404; *p* = 0.017.

**Figure 4.**
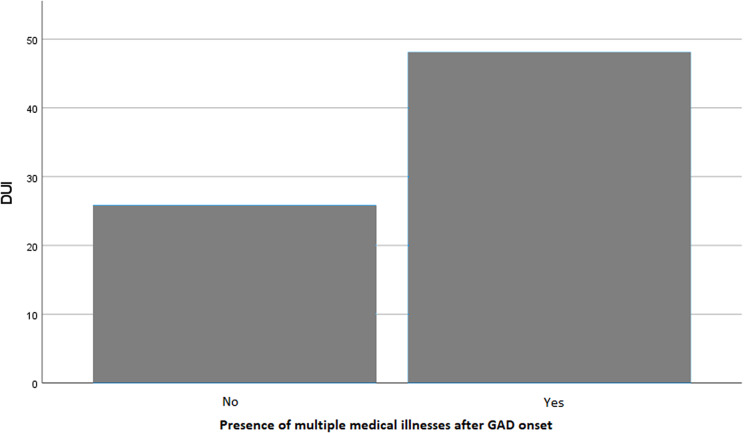
Trend toward significance in the association between DUI and the presence of multiple medical illnesses after GAD onset. Abbreviation: DUI, duration of untreated illness. Statistics: *B* = 55.778; *p* = 0.076.

Variables that were statistically significant in univariate analyses were included as independent factors in a linear multivariate regression model, with DUI length as the dependent variable. The model was deemed reliable according to the Durbin–Watson test (1.992). Earlier age at onset (*B* = −0.428; *p* = 0.023) (Figure 1), a longer duration of illness (*B* = 0.431; *p* < 0.001) (Figure 2), and the presence of multiple side effects (*B* = 55.778; *p* = 0.017) (Figure 3) were significantly associated with a longer DUI. Additionally, a trend toward statistical significance emerged for the association between multiple medical comorbidities developing after GAD onset and longer DUI (*B* = 13.122; *p* = 0.076) (Figure 4).

No further significant associations were found between DUI and the type of psychiatric comorbidity developing after GAD onset or the cluster of comorbid PD (*p* > 0.05).

## Discussion

The present study sought to identify sociodemographic and clinical factors associated with a longer DUI in GAD. Patients with a longer DUI had an earlier age at onset, a longer duration of illness, and experienced multiple side effects from pharmacological treatment. Additionally, a trend toward statistical significance emerged in the association between longer DUI and the development of multiple medical comorbidities after GAD onset. Univariate analyses indicated a positive relationship between longer DUI and the occurrence of post-onset panic disorder (compared to other post-onset comorbidities) and Cluster C PD (compared to other comorbid PDs or no PD); however, these findings were not confirmed in the final model.

The mean DUI in our sample was 31 months (±65.25), with a maximum of 408 months. This aligns with previous findings indicating that patients with anxiety disorders frequently delay several years before initiating appropriate pharmacological treatment.^27^^,^^28^ However, it should be noted that the mean DUI in our sample was considerably shorter compared to previous studies on GAD—for example, 153.36 ± 85.2 months,^25^ 87.07 ± 97.47 months,^23^ and 77.47 ± 95.76.^29^ While this discrepancy may be due to sample characteristics, it may also reflect the progressive reduction in treatment latency observed in psychiatric cohorts in recent years.^28^ The delay in seeking treatment in GAD patients may be explained by the disorder’s insidious onset and gradual functional impairment, which may lead affected individuals not to recognize the need for medical attention promptly. Notably, patients with panic disorder, who typically experience more acute and disabling symptoms at onset,^30^ tend to report a significantly shorter DUI.^23^^,^^24^ GAD patients may be more inclined to seek treatment when somatic symptoms emerge, turning to primary care physicians. However, somatic symptoms are frequently underrecognized in primary care, leading to extensive tests to rule out physical causes rather than addressing the underlying anxiety disorder.^31^ Even when GAD is diagnosed in this setting, it is commonly treated with benzodiazepines,^32^^,^^33^ which may provide temporary symptom relief but also delay the initiation of guideline-recommended pharmacological treatment. Another contributing factor is that anxiety symptoms are often mistaken for personality traits rather than recognized as manifestations of a treatable disorder.^34^ Finally, affected individuals often fear medication side effects, which may further hinder the timely management of GAD.^35^

Prior research has highlighted that a prolonged DUI may lead to an unfavorable illness course in GAD and other anxiety disorders, as evidenced by a longer duration of illness,^25^^,^^22^ reduced response to pharmacotherapy,^25^ and an increased frequency of post-onset depression.^21^ These findings are consistent with our results showing that a longer DUI is linked to both a prolonged illness duration and an increased risk of multiple side effects. It should be considered that prolonged anxiety can lead to alterations in the autonomic nervous system, increasing sensitivity to external stimuli, including the initiation of new pharmacological therapies.^36^^,^^37^ Early initiation of pharmacotherapy may, therefore, be associated with better tolerability and effectiveness due to less pronounced dysregulation of biological systems. Notably, the relation between shorter latency to pharmacotherapy and improved treatment response has been reported only for guideline-recommended therapies (e.g., SSRIs/SNRIs)—which are known to induce neural changes^38^—and not for benzodiazepines.^25^ In contrast, a longer DUI could reduce tolerability, increase the likelihood of poor compliance or treatment interruptions, and contribute to a chronic course of illness.

Our results indicate that patients with a longer DUI are more likely to develop comorbid medical conditions after the onset of GAD. Although this association has not been previously reported for anxiety disorders, increased rates of medical comorbidities have been documented in bipolar patients with a longer DUI.^39^^,^^40^ This relationship may reflect structural barriers to accessing medical care (e.g., lower socioeconomic income and geographical isolation), as well as attitudinal barriers, such as the belief that health issues will resolve without intervention or the fear of treatment-related adverse events.^41^ Additionally, untreated patients with GAD are likely to experience elevated stress levels, which may contribute to unhealthy lifestyle choices and increased cortisol production, heightening the risk of poor glucose tolerance and high blood pressure.^42^

Consistent with a previous study on GAD,^25^ our results indicate that an earlier age at onset is associated with a longer DUI. Of note, a similar inverse relationship has also been reported in patients with panic^22^ and mood disorders.^18^^,^^43^ One possible explanation for this trend is that younger individuals and their families may underestimate symptoms, interpreting them as typical emotional dysregulation of adolescence and young adulthood.^44^ This perception may act as a barrier to seeking treatment, potentially contributing to a poorer illness course.^44^

Finally, univariate analyses revealed that participants with a longer DUI were more likely to have a Cluster C or NOS PD, or to develop panic disorder, compared to other psychiatric comorbidities. Panic disorder may be considered an exacerbation of symptoms in individuals with untreated GAD, while the presence of PDs may represent an additional obstacle to accessing mental care, owing to avoidant-dependent traits or behavioral rigidity.^45^

Our findings should be considered in light of several limitations. First, the sample size could have been larger had additional mental healthcare facilities been included. Second, due to the cross-sectional design of our study, we are unable to provide prospective data on long-term outcomes. Moreover, we were unable to include data on illness severity and functional impact, as the retrospective nature of the study meant such information was either unavailable or inconsistently assessed across services using different evaluation tools. We chose to include only patients who had completed at least 3 months of treatment, as this duration is typically required to assess pharmacological response and tolerability; however, this criterion may have excluded individuals with more acute presentations. In addition, the sample size was insufficient to provide adequate power for subgroup analyses. Furthermore, most variables were analyzed dichotomously and then further stratified into specific subgroups; alternative approaches (eg, merging certain categories) may have allowed for more nuanced statistical exploration. Finally, the accuracy of the data extracted from medical records, such as illness onset, may have been affected by recall bias due to reliance on information provided by patients and family members.

Further studies with larger sample sizes and longitudinal designs are needed to confirm the present findings.

## Conclusions

Our findings support existing evidence that patients with anxiety disorders often experience significant delays in initiating appropriate pharmacological treatment.^27^^,^^28^ Individuals with an earlier age at onset may be particularly vulnerable in this regard, as young patients and their caregivers are more likely to overlook symptoms and defer help-seeking.^46^

A longer DUI in GAD appears to contribute to a more complicated illness course, with evidence suggesting that reducing treatment delays could improve disease management.^24^^,^^25^ The association between treatment delays and a worsening illness course is well-documented across a wide range of psychiatric disorders,^19^^,^^43^^,^^47^ emphasizing the need to implement prevention strategies. Further research is needed to confirm these findings and identify specific intervention targets to reduce DUI in patients with GAD.

## Data Availability

De-identified data will be made available by the corresponding author upon reasonable request.
